# Identification of genes regulated by lipids from seaweed Susabinori (*Pyropia yezoensis*) involved in the improvement of hepatic steatosis: Insights from RNA-Seq analysis in obese *db*/*db* mice

**DOI:** 10.1371/journal.pone.0295591

**Published:** 2023-12-12

**Authors:** Sayaka Iizasa, Koji Nagao, Keisuke Tsuge, Yukio Nagano, Teruyoshi Yanagita

**Affiliations:** 1 Graduate School of Science and Engineering, Kagoshima University, Kagoshima, Japan; 2 Department of Applied Biochemistry and Food Science, Saga University, Saga, Japan; 3 Industrial Technology Center of Saga, Saga, Japan; 4 Analytical Research Center for Experimental Sciences, Saga University, Saga, Japan; 5 Department of Health and Nutrition Science, Nishikyushu University, Saga, Japan; 6 Saga Foods & Cosmetics Laboratory, Division of Research and Development Promotion, Saga Prefectural Regional Industry Support Center, Saga, Japan; Tokyo University of Agriculture, JAPAN

## Abstract

Hepatic steatosis is an early stage in the progression of non-alcoholic fatty liver disease (NAFLD) and can lead to the development of non-alcoholic steatohepatitis (NASH), a major cause of liver-related morbidity and mortality. Identification of dietary components that can alleviate hepatic steatosis is crucial for developing effective therapeutic strategies for NAFLD. Recently, we demonstrated the impact of lipids extracted from the marine red alga Susabinori (*Pyropia yezoensis*) in a murine model of type 2-diabete (*db*/*db*). We found that Susabinori lipids (SNL), abundant in eicosapentaenoic acid (EPA)-containing polar lipids, protected against obesity-induced hepatic steatosis in *db*/*db* mice. To understand the specific genes or biological pathways underlying the effects of SNL, we conducted RNA-Seq analysis of the hepatic transcriptome. By performing comparative analysis of differentially expressed genes between normal mice and *db*/*db* mice consuming a control diet, as well as SNL-fed *db*/*db* mice, we identified the 15 SNL-dependent up-regulated genes that were down-regulated in *db*/*db* mice but up-regulated by SNL feeding. Gene ontology and pathway analysis on these 15 genes demonstrated a significant association with the metabolisms of arachidonic acid (AA) and linoleic acid (LA). Furthermore, we observed alterations in the expression levels of monoacylglycerol lipase (*Magl*) and fatty acid-binding protein 4 (*Fabp4*) in the SNL-fed *db*/*db* mice, both of which are implicated in AA and LA metabolism. Additionally, the livers of SNL-fed *db*/*db* mice exhibited reduced levels of AA and LA, but a high accumulation of EPA. In conclusion, the SNL diet might affect the metabolisms of AA and LA, which contribute to the improvement of hepatic steatosis. Our findings provide insights into the molecular mechanisms underlying the beneficial effects of SNL.

## Introduction

Hepatic steatosis constitutes an incipient stage in the evolution of non-alcoholic fatty liver disease (NAFLD), a hepatic manifestation of metabolic syndrome [1, 2]. Progression of NAFLD can lead to non-alcoholic steatohepatitis (NASH), a significant contributor to liver-related morbidity and mortality, marked by the presence of steatosis, inflammation, and fibrosis, which culminate in liver cirrhosis and cancer [1, 3, 4]. The identification of dietary components that alleviate hepatic steatosis holds great promise for the development of more efficacious and secure therapeutic strategies for NAFLD and its progression towards NASH.

Recently, we have investigated the impact of lipids extracted from the marine red alga Susabinori (*Pyropia yezoensis*, a scientific name change to *Neopyropia yezoensis* was proposed [5], but the scientific name *Pyropia yezoensis* has been proposed to be restored [6]) in type 2-diabetic model mice *db*/*db*, which possess a specific mutation of the *db* gene that encodes the leptin receptor [7]. Susabinori lipids (SNL) is a unique source of eicosapentaenoic acid (EPA)-containing polar lipid, primarily comprising EPA (62.4%) and palmitic acid (24.7%) [7]. We have demonstrated that feeding SNL to *db*/*db* mice protect the development of obesity-induced hepatic steatosis [7]. A four-week of SNL diet resulted in reduction of hepatic triglyceride accumulation and activity of hepatic injury marker in the serum of *db*/*db* mice. Moreover, it elicited a significant increase in serum levels of adiponectin, a hepatoprotective adipocytokines. Furthermore, the SNL diet altered the expression levels of key marker genes associated with lipid metabolism, inflammation, and anti-inflammation [7]. Based on these results and previous reports, we have proposed that EPA released from SNL effected on the increased secretion of adiponectin, thereby contributing to the amelioration of obesity-induced hepatic steatosis [7]. However, the choice of these appropriate markers to define the effect of SNL were based on several speculation or postulation derived from prior research. Thus, the specific genes or biological pathways regulated by SNL remain inadequately understood. Hence, this study aims to investigate the mechanisms of action of SNL in the liver of obese *db*/*db* mice through the comprehensive analysis of transcriptional changes using RNA-Seq.

RNA-Seq analysis conducted on livers of normal mice and *db*/*db* mice identified the 15 SNL-dependent up-regulated genes that were down-regulated in *db*/*db* mice but up-regulated by SNL feeding. We demonstrated a significant association between these 15 genes and the metabolisms of arachidonic acid (AA) and linoleic acid (LA). Our findings highlight the target genes of SNL diet and propose the mechanisms underlying the improvement of hepatic steatosis induced by SNL.

## Materials and methods

### Preparation and characterization of seaweed Susabinori lipid

Hot-air dried seaweed Susabinori (*Pyropia yezoensis*) was supplied from JA Saga (Saga, Japan). SNL used in our studies was extracted by the method of Folch *et al*. and Bligh and Dyer [8, 9], as described in detail previously [7]. Briefly, lipid extraction was performed using chloroform: methanol (V/V = 2:1) from dried Susabinori powder, and the chloroform layer was separated by adding 0.8% KCl. Chlorophyll and carotenoids were removed from the extract using a graphite carbon column, and the eluate was vacuum dried to obtain SNL. Characterization of the SNL samples was performed using thin-layer chromatography and gas chromatography-mass spectrometry (GC-MS), as described previously [7]. As a result, the SNL content obtained from 600 g of dried Susabinori powder was 26.5 g (4.4%), consisting mainly of phospholipids and glycolipids, with EPA (62.4%) as the main component of the fatty acid composition [7].

### Animals and diets

All aspects of the experiment were conducted according to the guidelines provided by the ethical committee for experimental animal care of Saga University (No. 19-002-1). Five-week-old male BKS.Cg- +Lepr^db^/+Lepr^db^/Jcl (*db/db*) mice and C57BL/6J mice were purchased from CLEA Japan Inc. (Tokyo, Japan). Mice were housed individually in plastic cages and kept at 24 °C on a 12 h light-dark cycle. After a 1-week adaptation period on a powder chow diet (CE-2, CLEA Japan Inc., Tokyo, Japan), *db/db* mice were randomly divided into two groups: a control diet group (control group, n = 6) and a SNL diet group (SNL group, n = 6). The SNL group was fed the control diet containing 2% of SNL. The C57BL/6J mice, the progenitors of *db/db* mice, were fed the control diet (normal group, n = 6). The mice consumed the diets using Rodent CAFÉ (KBT Oriental Co. Ltd., Saga, Japan) and were given water ad libitum for four weeks. The details of the diets have been previously published [7], and the amount of consumed food was measured daily for each mouse with no significant differences found in the total food intake between control and SNL groups.

At the end of the feeding period, mice were sacrificed by exsanguination from the heart under isoflurane anesthesia after a 9 h starvation time interval. Livers and other organs, including brain, heart, spleen, kidney, adrenal grand, testis, muscle, and white adipose tissue from perirenal were excised immediately, serum was separated from the blood. The tissue and serum were immediately frozen in liquid nitrogen and stored at −80 °C until analysis.

### RNA-Seq sample preparation and analysis

Total RNA was extracted from 100 mg of liver using a RNeasy Lipid Tissue Mini Kit (Qiagen, Tokyo, Japan). RNA samples were sent to Novogene (Singapore) for RNA sample quality test, library construction, and sequencing on the Illumina NovaSeq 6000 S4 platform (n = 3, per group). By removing the reads containing poly-N, adapter, and low-quality using cutadapt v 1.18 and PRINSEQ-lite v 0.20.4, clean data (clean reads) were obtained and the paired-end reads were aligned to mouse reference genome (GRCm38) using hisat2 v2.1.0. The transcripts were assembled using Cufflinks v.2.2.1. The read counts of each data set were obtained by HTSeq-count v.0.11.2. By analyzing the read counts, the differentially expressed genes (DEGs) were calculated with R packages TCC v1.10.0 [10], which use edgeR v3.12.1 [11] internally. The RNA-Seq raw data used in this paper have been deposited in the DNA Data Bank of Japan (DDBJ) Sequence Read Archive (DRA) (accession no. DRA016628).

### Gene ontology (GO) enrichment analysis and pathway analysis

The GO enrichment analysis and pathway analysis for SNL-dependent up- or down-regulated genes was performed using Functional Annotation Charts of DAVID Bioinformatics Resources 6.8 (https://david.ncifcrf.gov/) [12]. Pathway analysis uses the KEGG databases (https://www.genome.jp/kegg/) [13]. Only GO terms and pathways with P-value < 0.05 were listed.

### Quantitative real-time PCR (qRT-PCR) analysis

After the total RNA isolation as above, reverse transcription was performed using 0.2 μg of total RNA using ReverTra Ace^®^ qPCR RT Master Mix with gDNA Remover (Toyobo, Osaka, Japan) according to the manufacturer’s protocol. qRT-PCR was run on a Thermal Cycler Dice^®^ RealTime System TP800 (Takara Bio Inc, Shiga, Japan) and THUNDERBIRD^®^ SYBR qPCR Mix (Toyobo, Osaka, Japan) with the following conditions: 1 cycle of 30 s at 95 °C, and 40 cycles of 5 s at 95 °C and 30 s at 60 °C. *β-actin* was used as an internal standard. The sequences of gene-specific primers are mentioned in S1 Table. Each experiment was repeated at least three times.

### Analysis of fatty acid contents in organs and serum by GC-MS

Lipids contained in the organs and serum were extracted and purified from 6 mice of each group by the method of Bligh and Dyer [9]. GC-MS conditions were as follows: equipment, GC2010 + QP2010 (Shimadzu Corp., Tokyo, Japan); column, SP2380 (L 100 m × I.D. 0.25 mm × d 0.2 μm, Sigma-Aldrich Co., Tokyo, Japan); carrier gas flow rate, 20 cm/s (helium); column temperature, 140 °C for 5 min, 140–205 °C at 1.5 °C/ min, 205–240 °C at 10 °C/min, 240 °C for 8 min; injection, 1 μL; split ratio 20:1 at 250 °C; ion source, electron ionization. Samples and standards were treated with 0.5 M potassium hydroxide-methanol solution (100 °C for 9 min) followed by 10% BF3-methanol solution (100 °C for 7 min) to derivatize into fatty acid methyl esters prior to GC-MS.

### Use of generative AI

ChatGPT (https://chat.openai.com/) was used for rewriting into more appropriate English and for English text proofreading. Afterward, the correctness of the revisions was confirmed by the authors.

## Results

### Identification of SNL-dependent up- and down-regulated genes in obese *db*/*db* mice

To investigate the impact of SNL on the gene expression patterns in the liver of obese *db*/*db* mice, we attempted to distinguish SNL-dependent up- and down-regulated genes through RNA-Seq analysis. Total mRNA was extracted from the livers of C57BL/6J mice maintained on a control diet (designated as the normal group) and *db*/*db* mice either consuming a control diet (termed the control group) or an SNL diet (designated as the SNL group). Subsequently, the resultant RNA-Seq datasets were comparatively analyzed. It should be noted that there were no significant differences in the total food intake between the control and SNL groups, suggesting that the changes in gene expression between them were due to SNL in the diet [7].

**Fig 1:**
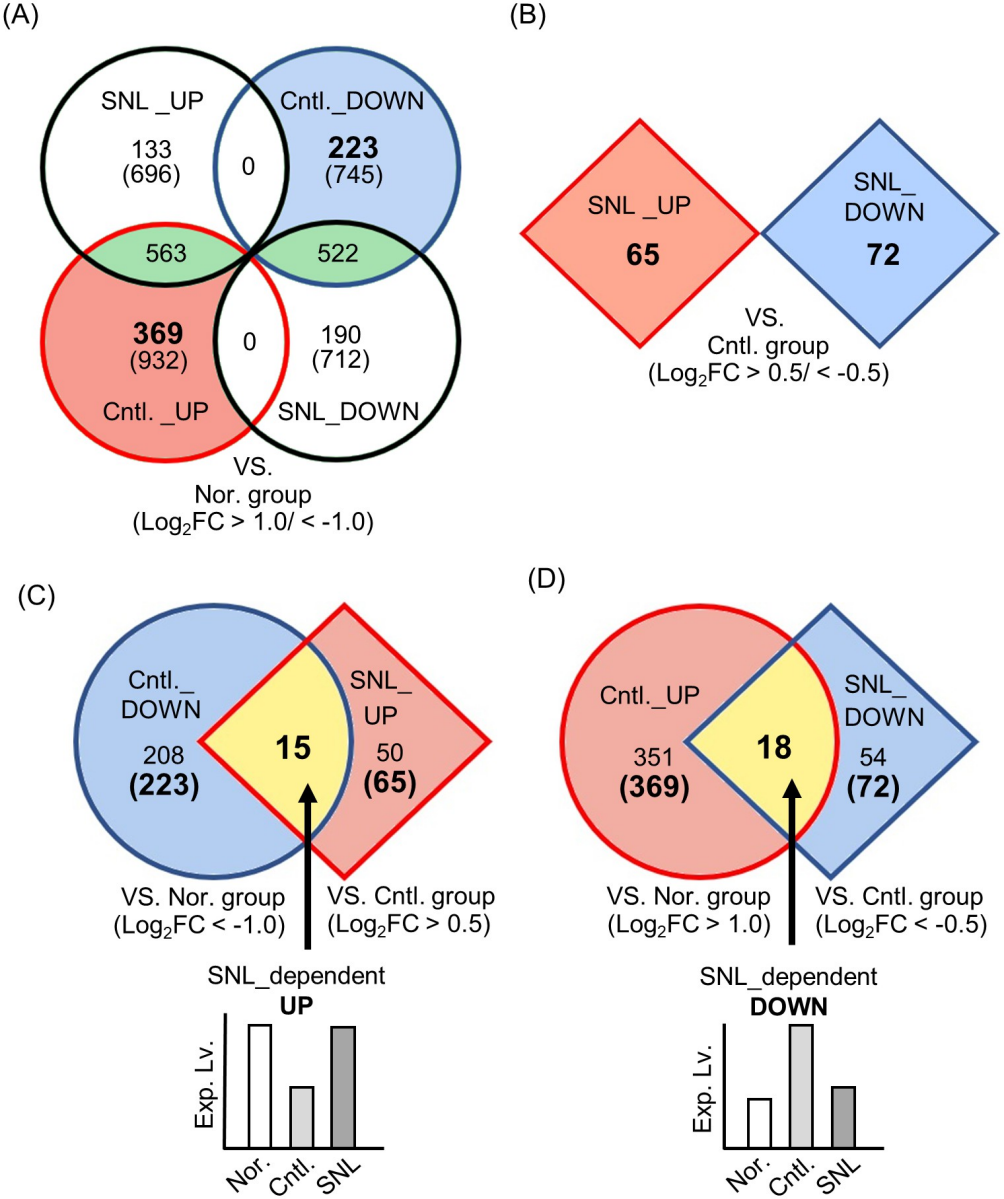
Identification of SNL-dependent up- or down-regulated genes. (A) Venn diagram demonstrating the number of DEGs in the control and the SNL groups compared with the normal group (Log_2_FC > 1.00, Log_2_FC < -1.00). The numbers in the parentheses indicate the number of genes identified as up- or down-regulated in each group. The numbers in green area indicates the number of SNL-unresponsive genes. (B) The number of DEGs in the SNL groups compared with the control group (Log_2_FC > 0.50, Log_2_FC < -0.50). (C and D) The number of DEGs obtained from (A) and (B) were compared to identify the SNL-dependent up- or down-regulated genes (each yellow areas). An example bar graph illustrates the changes of gene expression levels. All genes with a false discovery rate (FDR) < 0.05 were classified as DEGs.

To examine the effects of SNL rather than the influence of genetic differences between *db*/*db* mice and normal mice, we conducted the following analysis. An analysis of the DEGs between the normal and control groups revealed that 932 genes were up-regulated in the control group (FDR; false discovery rate < 0.05, Log_2_FC; Log fold change > 1.00) (Fig 1A, red circle), while 745 genes were down-regulated (FDR < 0.05, Log_2_FC < -1.00) (Fig 1A, blue circle). On the other hand, analysis of the DEGs between the normal and the SNL groups revealed that 696 genes were up-regulated and 712 genes were down-regulated (Fig 1A, black circles). However, the DEGs identified in both the control and SNL groups shared a common set of 563 up-regulated and 522 down-regulated genes (Fig 1A, green areas). Given that these common DEGs may result from a mutation in the leptin receptor gene in *db*/*db* mice, these genes are "SNL-unresponsive genes". To investigate the effect of SNL on gene expression, we excluded these SNL-unresponsive genes from the 932 up- and 745 down-regulated genes in the control group and focused on the remaining 369 up-regulated and 223 down-regulated genes (Fig 1A, red and blue area, respectively).

In addition, the RNA-Seq data obtained from the SNL group was compared to that from the control group. In order to identify a larger number of candidate gene associating with SNL diet, we analyzed with cutoff values of |Log_2_FC|> 0.50 in this analysis (FDR < 0.05). The transcript levels of 65 genes were up-regulated in the SNL group (Log_2_FC > 0.50) (Fig 1B, red diamond) and 72 genes were down-regulated (Log_2_FC < -0.50) (Fig 1B, blue diamond). We compared these 65 up-regulated genes from SNL group with 223 down-regulated genes from control group, and regarded the 15 genes commonly identified in both groups as SNL-dependent up-regulated genes (Fig 1C, yellow area). Likewise, 18 genes also identified as SNL-dependent down-regulated genes by comparing the 72 down-regulated genes from SNL group with 369 up-regulated genes from control group (Fig 1B, yellow area).

We also conducted qRT-PCR for randomly selected SNL-responsive genes to validate our RNA-Seq data. For all the tested genes, transcript levels determined by qRT-PCR were similar to those detected using RNA-Seq, indicating the reliability of our RNA-Seq data (S1 Fig).

### Characterization of 15 SNL-dependent up-regulated genes

Table 1 shows details of the 15 SNL-dependent up-regulated genes. Among them, prostaglandin D2 synthase (*Ptgds*) showed the highest Log_2_FC value (3.15). Other notable features include elevated gene expression of 3 cytochrome P450 (Cyp) 2c subfamily genes (*Cyp2c37*, *Cyp2c50*, and *Cyp2c54*). Of these three, *Cyp2c37* and *Cyp2c54* had Log_2_FC value above 1.00.

**Table 1:** SNL dependent up-regulated 15 genes in liver of *db/db* mice.

Gene	Description	Log_2_FC	FDR
*Ptgds*	Prostaglandin D2 synthase	3.1497	1.09 x 10^−14^
*Cyp2c37*	Cytochrome P450, family 2. subfamily c, polypeptide 37	1.6133	7.93 x 10^−7^
*Cyp2c54*	Cytochrome P450, family 2, subfamily c, polypeptide 54	1.1956	4.42 x 10^−6^
*Hal*	Histidine ammonia lyase	1.1927	2.91 x 10^−5^
*Ces1b*	Carboxylesterase 1B	1.0636	1.40 x 10^−3^
*Klf15*	Kruppel-like factor 15	0.9581	2.30 x 10^−3^
*Fam234b*	Family with sequence similarity 234, member B	0.9233	1.80 x 10^−4^
*Pigr*	Polymeric immunoglobulin receptor	0.8899	6.62 x 10^−7^
*Cyp2c50*	Cytochrome P450, family 2, subfamily c, polypeptide 50	0.8884	4.10 x 10^−4^
*Ces2a*	Carboxylesterase 2A	0.8705	1.74 x 10^−3^
*Amdhd1*	Amidohydrolase domain containing 1	0.8171	5.81 x 10^−5^
*Etnk2*	Ethanolamine kinase 2	0.8084	7.19 x 10^−5^
*2810459M11Rik*	RIKEN cDNA 2810459M11 gene	0.7037	3.32 x 10^−3^
*Scarb1*	Scavenger receptor class B, member 1	0.5574	2.79 x 10^−3^
*Gpld1*	Glycosylphosphatidylinositol specific phospholipase D1	0.5435	1.75 x 10^−2^

Genes, which down-regulated in liver of *db/db* mice but up-regulated by SNL diet, were identified. The Log_2_FC and P-Value shown in Table were obtained from the result of DEGs investigation between control and SNL groups (FDR < 0.05, Log_2_FC > 0.50).

We performed GO analysis of these 15 genes using Functional Annotation Chart generated by the DAVID web interface (Table 2). The classification of Biological Process (BP) GO analysis revealed 7 GO terms, including 4 lipid metabolism-related terms and 3 histidine-related terms (P < 0.05, Table 2). *Cyp2c* subfamily genes were identified to be responsible for the enrichment of 3 lipid metabolism-related terms: "Epoxygenase P450 pathway", "Linoleic acid metabolic process", and "Arachidonic acid metabolic process". Glycosylphosphatidylinositol specific phospholipase D1 (*Gpld1*) and scavenger receptor class B member 1 (*Scarb1*) were responsible for the enrichment of "Positive regulation of triglyceride biosynthetic process”. Whereas amidohydrolase domain containing 1 (*Amdhd1*) and histidine ammonia lyase (*Hal)* were responsible for the enrichment of 3 terms related to histidine.

**Table 2:** BP, CC, and MF GO-term-enriched tables of SNL dependent up-regulated 15 genes.

Term		GeneRatio (%)	P-value	Genes
**BP**	Epoxygenase P450 pathway	20.0	1.45 x 10^−4^	*Cyp2c37*, *Cyp2c54*, *Cyp2c50*
	Histidine catabolic process to glutamate and formamide	13.3	2.43 x 10^−3^	*Amdhd1*, *Hal*
	Histidine catabolic process to glutamate and formate	13.3	2.43 x 10^−3^	*Amdhd1*, *Hal*
	Histidine metabolic process	13.3	3.04 x 10^−3^	*Amdhd1*, *Hal*
	Linoleic acid metabolic process	13.3	6.67 x 10^−3^	*Cyp2c54*, *Cyp2c50*
	Positive regulation of triglyceride biosynthetic process	13.3	0.01030	*Gpld1*, *Scarb1*
	Arachidonic acid metabolic process	13.3	0.02169	*Cyp2c54*, *Cyp2c50*
**CC**	Intracellular membrane-bounded organelle	33.3	8.18 x 10^−4^	*Cyp2c37*, *Cyp2c54*, *Cyp2c50 Gpld1*, *Scarb1*
	Organelle membrane	20.0	1.45 x 10^−3^	*Cyp2c37*, *Cyp2c54*, *Cyp2c50*
	Extracellular space	33.3	0.01025	*Ptgds*, *Gpld1*, *Ces2a*, *Pigr*, *Ces1b*
	Endoplasmic reticulum	26.7	0.04230	*Cyp2c37*, *Cyp2c54*, *Ptdgs*, *Cyp2c50*
**MF**	Aromatase activity	20.0	3.18 x 10^−4^	*Cyp2c37*, *Cyp2c54*, *Cyp2c50*
	Arachidonic acid epoxygenase activity	20.0	5.44 x 10^−4^	*Cyp2c37*, *Cyp2c54*, *Cyp2c50*
	Steroid hydroxylase activity	20.0	7.18 x 10^−4^	*Cyp2c37*, *Cyp2c54*, *Cyp2c50*
	Oxidoreductase activity, acting on paired donors, with incorporation or reduction of molecular oxygen	20.0	2.39 x 10^−3^	*Cyp2c37*, *Cyp2c54*, *Cyp2c50*
	Monooxygenase activity	20.0	2.83 x 10^−3^	*Cyp2c37*, *Cyp2c54*, *Cyp2c50*
	Linoleic acid epoxygenase activity	13.3	2.98 x 10^−3^	*Cyp2c54*, *Cyp2c50*
	Heme binding	20.0	7.26 x 10^−3^	*Cyp2c37*, *Cyp2c54*, *Cyp2c50*
	Iron ion binding	20.0	0.01050	*Cyp2c37*, *Cyp2c54*, *Cyp2c50*

The 15 SNL dependent up-regulated genes were classified by functional categories under the following GO terms (P < 0.05): biological process (BP), cellular component (CC), and molecular function (MF), using Functional Annotation Charts generated by DAVID Bioinformatics Resources 6.8. GeneRatio indicates the percentages of involved genes/total genes.

Furthermore, Cellular Component (CC) GO analysis showed that approximately 50% of target genes were significantly enriched in membrane-related GO terms, such as “Intracellular membrane-bounded organelle” and “Organelle membrane” (P < 0.05, Table 2). Moreover, Molecular Function (MF) GO analysis classified target genes via 8 GO terms, including “Arachidonic acid epoxygenase activity” and “Linoleic acid epoxygenase activity” (P < 0.05, Table 2). Interestingly, only 3 genes, *Cyp2c37*, *Cyp2c54*, and *Cyp2c50*, were responsible for the enrichment of all MF GO terms.

Additionally, KEGG pathway analysis indicated that the target genes involved in 9 pathways, including “Arachidonic acid metabolism”, “Linoleic acid metabolism”, and “Metabolic pathways” (P < 0.05, Table 3). Notably, three *Cyp2c* subfamily genes were also responsible for the enrichment of 8 pathways among these 9 pathways.

**Table 3:** Pathway enrichment analysis of SNL dependent up-regulated 15 genes.

Term	GeneRatio (%)	P-value	Genes
Arachidonic acid metabolism	26.7	1.70 x 10^−4^	*Cyp2c37*, *Cyp2c54*, *Ptgds*, *Cyp2c50*
Linoleic acid metabolism	20.0	1.80 x 10^−3^	*Cyp2c37*, *Cyp2c54*, *Cyp2c50*
Metabolic pathways	46.7	2.29 x 10^−3^	*Amdhd1*, *Cyp2c37*, *Cyp2c54*, *Ptgds*, *Hal*, *Etnk2*, *Cyp2c50*
Steroid hormone biosynthesis	20.0	5.37 x 10^−3^	*Cyp2c37*, *Cyp2c54*, *Cyp2c50*
Retinol metabolism	20.0	5.61 x 10^−3^	*Cyp2c37*, *Cyp2c54*, *Cyp2c50*
Chemical carcinogenesis	20.0	5.98 x 10^−3^	*Cyp2c37*, *Cyp2c54*, *Cyp2c50*
Inflammatory mediator regulation of TRP channels	20.0	0.01100	*Cyp2c37*, *Cyp2c54*, *Cyp2c50*
Serotonergic synapse	20.0	0.01202	*Cyp2c37*, *Cyp2c54*, *Cyp2c50*
Histidine metabolism	13.3	0.02952	*Amdhd1*, *Hal*

A P-Value < 0.05 was used as a threshold to select significant pathways. GeneRatio indicates the percentages of involved genes/total genes.

### Characterization of 18 SNL-dependent down-regulated genes

The SNL-dependent 18 down-regulated genes included 5 binding proteins: three S100 calcium binding proteins (*S100a10*, *S100a11*, and *S100g*), lectin galactose binding soluble 3 (*Lgals3*), and ras-related GTP binding D (*Rragd*) (Table 4). The most considerably down-regulated gene was activating transcription factor 3 (*Atf3*), exhibiting the lowest Log_2_FC value (-2.74). Furthermore, guanylate cyclase 2c (*Gucy2c*) and S100 calcium-binding protein G (*S100g*) also exhibited Log_2_FC values below -2.00. The BP GO analysis of these 18 genes revealed that chemokine (C-C motif) ligand 2 (*Ccl2*) and *Lgals3* were responsible for the enrichment of all 5 GO terms (P < 0.05, Table 5). Among these, 4 GO terms related to the chemotaxis of inflammation-related cells, including eosinophil, macrophage, monocyte, and neutrophil. The CC and MF GO analyses revealed only 3 extracellular-related GO terms and 2 binding-related terms, respectively (P < 0.05, Table 5). Despite performing a KEGG pathway analysis on these 18 genes, none of the pathways were statistically significant (P < 0.05). These findings suggest that, although the SNL-dependent down-regulated genes displayed the enrichment of some GO terms owing to the presence of genes with the similar annotation, they did not significantly contribute to any specific pathway. Hence, in this study, we focused on 15 SNL-dependent up-regulated genes for further examination.

**Table 4:** SNL dependent down-regulated 18 genes in liver of *db/db* mice.

Gene	Description	Log_2_FC	FDR
*Atf3*	Activating transcription factor 3	-2.7416	7.72 x 10^−9^
*Gucy2c*	Guanylate cyclase 2c	-2.5822	4.20 x 10^−3^
*S100g*	S100 calcium binding protein G	-2.27789	1.03 x 10^−2^
*Ccl2*	Chemokine (C-C motif) ligand 2	-1.6067	1.13 x 10^−2^
*Slc25a24*	Solute carrier family 25 (mitochondrial carrier, phosphate carrier), member 24	-1.5039	1.13 x 10^−2^
*Rragd*	Ras-related GTP binding D	-1.4842	2.66 x 10^−3^
*Tmem136*	Transmembrane protein 136 (Tlcd5)	-1.4169	2.61 x 10^−2^
*S100a11*	S100 calcium binding protein A11 (Emap1)	-1.3434	1.94 x 10^−3^
*RP23-350G1*.*5*	RP23-350G1.5	-1.3252	1.70 x 10^−7^
*Lgals3*	Lectin, galactose binding, soluble 3	-1.2422	2.79 x 10^−3^
*Klf6*	Kruppel-like factor 6	-1.2388	4.03 x 10^−6^
*Cgref1*	Cell growth regulator with EF hand domain 1	-1.2203	4.32 x 10^−2^
*2010003K11Rik*	RIKEN cDNA 2010003K11 gene	-1.1887	2.50 x 10^−4^
*Col5a2*	Collagen, type V, alpha 2	-1.1036	4.59 x 10^−2^
*Rarres1*	Retinoic acid receptor responder (tazarotene induced) 1	-0.8747	9.61 x 10^−3^
*Rhoq*	Ras homolog family member Q	-0.6948	1.27 x 10^−2^
*S100a10*	S100 calcium binding protein A10 (calpactin)	-0.6200	3.10 x 10^−2^
*Cstb*	Cystatin B	-0.5470	3.98 x 10^−2^

Genes, which up-regulated in liver of *db/db* mice but down-regulated by SNL diet, were identified. The Log_2_FC and P-Value shown in Table were obtained from the result of DEGs investigation between control and SNL groups (FDR < 0.05, Log_2_FC < -0.50).

**Table 5:** BP, CC, and MF GO-term-enriched tables of SNL dependent down-regulated 18 genes.

Term		GeneRatio (%)	P-value	Genes
**BP**	Eosinophil chemotaxis	11.1	7.17 x 10^−3^	*Ccl2*, *Lgals3*
	Macrophage chemotaxis	11.1	0.010022	*Ccl2*, *Lgals3*
	Positive regulation of calcium ion import	11.1	0.014998	*Ccl2*, *Lgals3*
	Monocyte chemotaxis	11.1	0.028389	*Ccl2*, *Lgals3*
	Neutrophil chemotaxis	11.1	0.048503	*Ccl2*, *Lgals3*
**CC**	Extracellular matrix	16.7	0.023289	*Ibsp*, *Lgals3*, *Col5a2*
	Extracellular space	27.8	0.029505	*Ibsp*, *Ccl2*, *Lgals3*, *Cstb*, *S100a11*
	Extracellular region	27.8	0.048065	*Ibsp*, *Cgref1*, *Ccl2*, *Lgals3*, *Col5a2*
**MF**	Calcium ion binding	27.8	1.37 x 10^−3^	*Cgref1*, *Slc25a24*, *S100a11*, *S100g*, *S100a10*
	GTP binding	16.7	0.031957	*Rhoq*, *Rragd*, *Gucy2c*

The 18 SNL dependent down-regulated genes were classified by functional categories under the following GO terms (P > 0.05): biological process (BP), cellular component (CC), and molecular function (MF) using the Functional Annotation Charts generated by DAVID Bioinformatics Resources 6.8. GeneRatio indicates the percentages of involved genes/total genes.

### Confirmation of SNL-induced up-regulation of arachidonic acid and linoleic acid metabolism-related genes; *Cyp2c37*, *Cyp2c50*, *Cyp2c54*, and *Ptgds*

**Fig 2:**
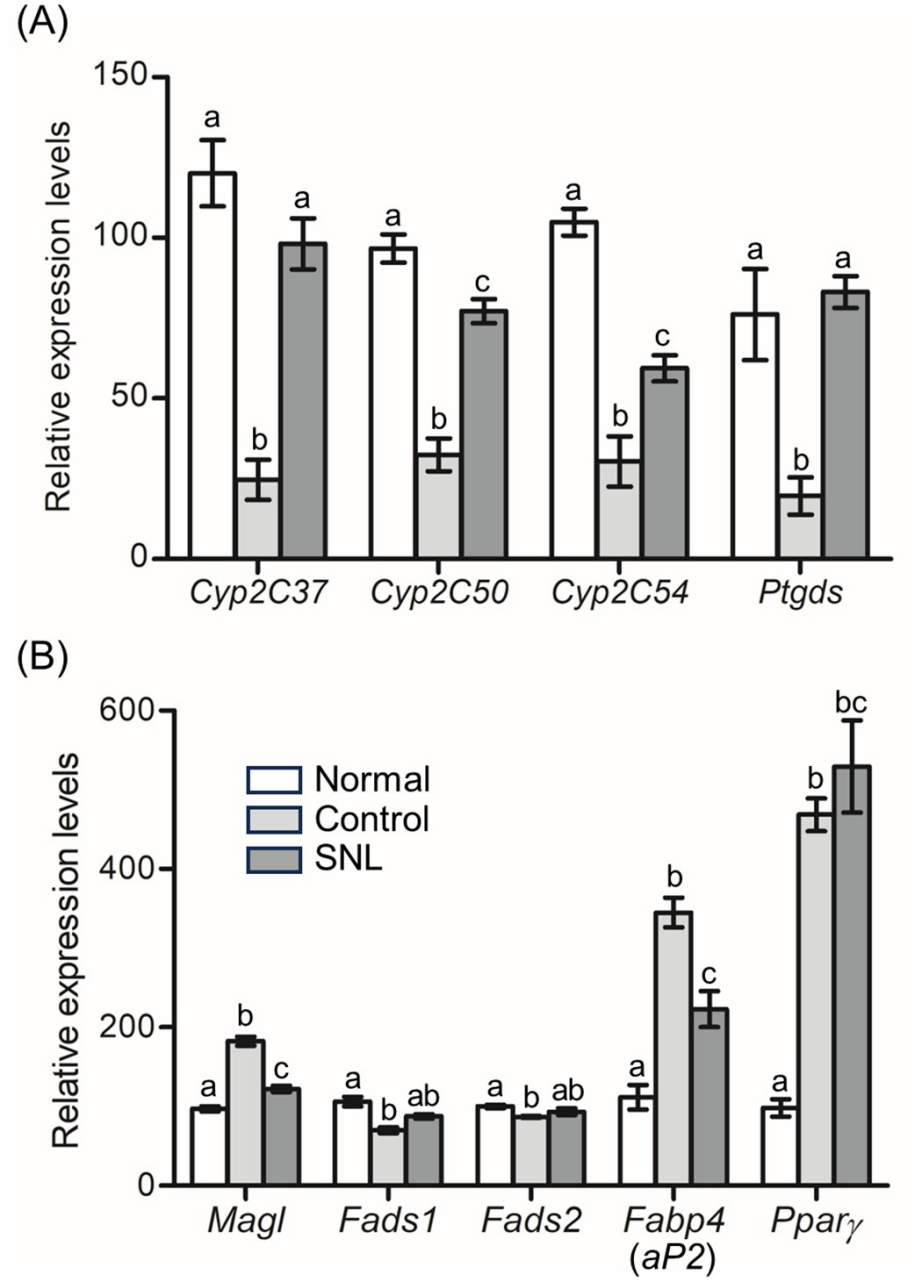
qRT-PCR analysis of AA and LA metabolism-related genes. (A) Among the 15 SNL-dependent up-regulated genes, the expression levels of *Cyp2c37*, *Cyp2c50*, *Cyp2c54*, and *Ptgds* were analyzed by qRT-PCR. (B) The expression levels of the 5 AA and LA metabolism-related genes were analyzed by qRT-PCR. The mean values were calculated from the results of three independent experiments. Means ± standard errors are presented. Significant differences among the means were determined by one-way ANOVA followed by Tukey’s Multiple Comparison Test. ^abc^ Different letters showed significant difference at *P* < 0.05.

Our KEGG analysis revealed a significant association between *Cyp2c37*, *Cyp2c50*, *Cyp2c54*, and *Ptgds* with the metabolisms of arachidonic acid (AA; 20:4ω-6) and linoleic acid (LA; 18:2ω-6) (Table 3). We regarded *Cyp2c37*, *Cyp2c50*, *Cyp2c54*, and *Ptgds* as AA and LA metabolism-related genes, and examined the expression levels of them by qRT-PCR to confirm the results of our RNA-Seq analysis. Results showed that the expression levels of all 4 genes were significantly down-regulated in the control groups higher in the SNL group compared to the control group (Fig 2A). Notably, the expression levels of *Cyp2c37* and *Ptgds* did not differ significantly between the SNL and normal groups, suggesting that the SNL diet effectively restored their expression levels in *db*/*db* mice to the same level as normal mice.

### The effect of SNL diet on the expression levels of AA and LA metabolism-related genes

Above results implied the further effect of SNL diet on the expression levels of cytosolic phospholipase A_2_ (*cPla*_*2*_), cyclooxygenase 1 (*Cox1*), and *Cox2*, which also play an important role in AA metabolism. However, the expression levels of these 3 genes were too low to be detectable by qRT-PCR in all 3 groups. This suggests that while metabolism of AA and LA in liver may be a target of the SNL diet, the effect is not accompanied by the alterations in the expression levels of *cPla*_*2*_, *Cox1*, and *Cox2*.

Subsequently, we focused on the expression levels of monoacylglycerol lipase (*Magl*), fatty acid desaturase 1 (*Fads1*), and *Fads2*, as AA and LA metabolism-related genes. MAGL regulates the metabolic pathway of AA by releasing AA as a degradation product of the 2-arachidonoylglycerol, one of well-established endocannabinoids. *Fads1* encoding Δ5-desaturase (D5D) and *Fads2* encoding Δ6-desaturase (D6D) catalyze the desaturation steps in the synthesis of AA from LA; LA is first desaturated by D6D to yield γ-LA (GLA; 18:3ω-6), then GLA is elongated to dihomo-GLA (DGLA; 20:3ω-6), which is converted to AA by D5D. Our results revealed a significant up-regulation of *Magl* and down-regulation of *Fads1* and *Fads2* in the control groups, as compared to the normal group (Fig 2B). The expression level of *Magl* was significantly decreased in the SNL group compared to the control group. On the other hand, both *Fads1* and *Fads2* were slightly elevated in the SNL groups compared to the control groups, but the differences between groups did not reach statistical significance (Fig 2B).

Furthermore, we examined the expression levels of peroxisome proliferator-activated receptor γ (*Pparγ*) and its transcriptional target gene fatty acid-binding protein 4 (*Fabp4*, also known as *aP2*). FABP4 bind directly to both AA and LA, and delivers these ligands from the cytosol to the nuclear receptor PPARγ. We found the up-regulation of both *Fabp4* and *Pparγ* in the control groups compared to the normal groups (Fig 2B). Although a reduction in *Fabp4* expression was observed in the SNL group compared to the control group, there were no significant differences in the expression levels of *Pparγ* between the SNL and control groups (Fig 2B).

### The effect of SNL diet on the fatty acid contents in liver of *db*/*db* mice

**Fig 3:**
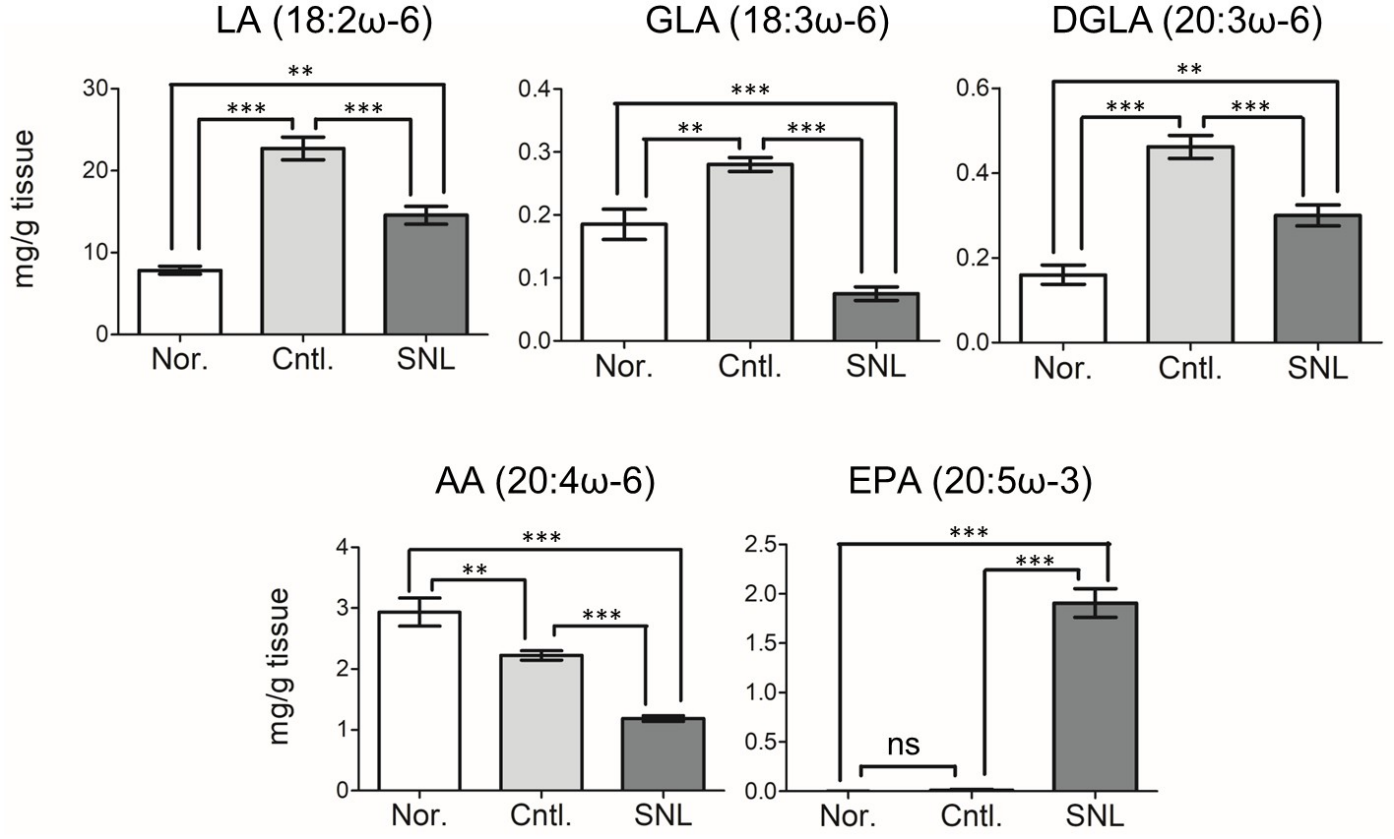
Comparison of liver fatty acid contents. The contents of hepatic LA, GLA, DGLA, AA, and EPA were measured (n = 6). Means ± standard errors are presented. Significant differences among the means were determined by one-way ANOVA followed by Tukey’s Multiple Comparison Test. *P < 0.05, **P < 0.01, ***P < 0.001.

To examine the association between the changes in expression levels of AA and LA metabolism-related genes and the fatty acid contents in liver, we assessed the hepatic content of LA, GLA, DGLA, and AA (Fig 3). The control group displayed elevated levels of LA, GLA, and DGLA, but these 3 fatty acids showed a significant decrease in the SNL group. Conversely, AA level was reduced in the control group compared to the normal group (but not significantly different), and the SNL group demonstrated even lower AA levels than those found in the control group. These results suggest that although the SNL diet could reduce liver content of LA, GLA, and DGLA, its effect on AA levels differed.

Furthermore, we assessed the hepatic content of EPA (20:5ω-3), which is the principal fatty acid constituent of SNL (62.4%) (Fig 3) [7]. The remarkably high accumulation of EPA was found only in the SNL group (Fig 3). Interestingly, not just in the liver, but also in the serum and other organs of SNL-treated mice, including the brain, heart, spleen, kidney, adrenal gland, testis, muscle, and white adipose tissue, an augmented accumulation of EPA was observed (S2 Table). These findings suggest that the SNL diet for 4 weeks could enhance the systemic and tissue concentrations of EPA.

## Discussion

Our primary interest is in revealing the genes or pathways as SNL targets by a study which was not made through any speculation or postulation but rather through a comprehensive analysis using RNA-Seq. We revealed 15 SNL-dependent up-regulated genes, which associate with AA and LA metabolisms. SNL-fed *db*/*db* mice exhibited a down-regulation of *Magl* and *Fabp4*, as well as a decrease in hepatic levels of LA, GLA, DGLA, and AA, but an increase in EPA. Numerous studies confirm the competitive interactions between AA and ω-3 polyunsaturated fatty acids (ω-3 PUFAs), including EPA and DHA [14–16]. We consider the possibility that the effect of competition between AA (and LA) metabolisms and EPA released form SNL in liver may relate to the changes of gene expression and contribute to the improvement of hepatic steatosis.

**Fig 4:**
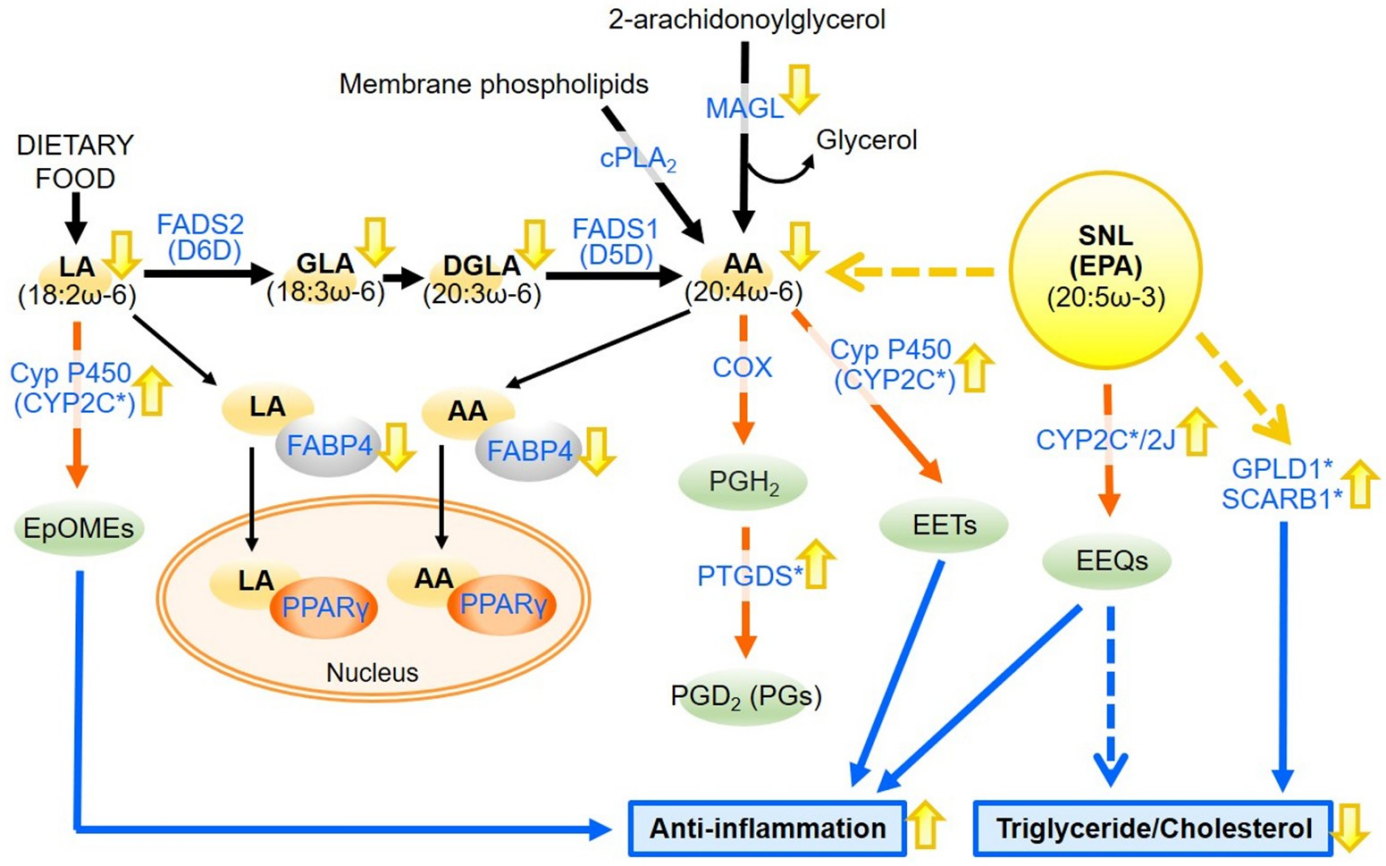
The effect of SNL on the liver of *db*/*db* mice. The effect of SNL on the pathways of AA and LA metabolisms and other pathways associated with the identified genes were illustrated. The blue font highlights proteins analyzed in this study. Of these, proteins encoded by SNL-dependent up-regulated genes are marked with an asterisk (CYP2Cs, PTGDS, GPLD1, and SCARB1). The effect of SNL on the expression levels of genes or lipid contents in the liver of *db*/*db* mice were indicated in yellow arrows. The orange arrows indicate the pathways of eicosanoids or epoxides biosynthesis from AA, LA, and SNL (EPA). The blue arrows indicate the proposed connection between the transcriptomic changes induced by SNL diet, as identified in this study, the phenotypic alterations observed in SNL-fed *db*/*db* mice [7].

Among of 15 genes, especially, we regarded *Cyp2c37*, *Cyp2c50*, *Cyp2c54*, and *Ptgds* as AA and LA metabolism-related genes. Notably, 3 *Cyp2c* subfamily genes were found to be responsible for the enrichment of 8 out of 9 identified pathways, suggesting their significant impact on SNL-induced alterations. In addition, *Ptgds* is the most dramatically up-regulated gene by SNL. These 4 genes are known as the key enzymes involved in the biosynthesis of eicosanoids, including epoxyeicosatrienoic acids (EETs), prostaglandins (PGs), and epoxyoctadecamonoenoic acids (EpOMEs), from AA or LA (Fig 4). *Cyp2c* subfamily genes are known as epoxygenases, that generate EETs and EpOMEs from free AA or LA, respectively [17–19] (Fig 4). *Ptgds* catalyzes the formation of PGD_2_ from AA-derived PGH_2_, the common precursor of all PGs (Fig 4). Interestingly, previous studies have reported the association of eicosanoids not only with NAFLD but also with diabetes and obesity, which are closely linked to hepatic steatosis [20–22]. Furthermore, other epoxygenase *Cyp2j3* gene delivery in *db*/*db* mice *in vivo* increased EETs levels and reversed insulin resistance, which was determined by plasma glucose levels, homeostasismodel assessment, insulin resistance index, and glucose tolerancetest [23]. Interestingly, CYP2C and CYP2J isoforms can epoxidate EPA and DHA to epoxyeicosatetraenoic acids (EEQs) and epoxydocosapentaenoic acids (EDPs), respectively [24]. We suggest that *Cyp2c37*, *Cyp2c50*, and *Cyp2c54* may be the key enzymes in the EEQs synthesis from the dietary EPA in liver (Fig 4).

Moreover, we postulate that the SNL-induced up-regulation of these 3 *Cyp2c* subfamily might change the levels of eicosanoids and epoxides, resulting in the reduction of inflammation. In general, CYP2C or CYP2J-derived EETs play a pivotal role in anti-inflammatory and anti-apoptotic mechanisms that protect against cell and organ injury (Fig 4) [16, 25, 26]. Notably, the overexpression of CYP2J2 in FHD-mice not only elicited reduced inflammatory responses but also exhibited a significantly decreased in plasma triglyceride levels and liver lipid accumulation [27]. Furthermore, CYP2C-derived EpOMEs, EEQs, and EDPs are also known as important epoxides which reduce inflammation and autophagy in liver [28, 29]. In fact, our research has revealed that SNL-fed *db*/*db* mice exhibited a significant decrease in the levels of hepatic triglyceride and the expression of inflammatory marker gene monocyte chemotactic protein 1 (*Mcp1*), also known as *Ccl2*, which is one of the 18 SNL-dependent down-regulated genes [7]. Our findings strongly suggest that the anti-inflammatory effect induced by SNL diet in the livers of *db*/*db* mice is likely attributed to the production of anti-inflammatory eicosanoids and epoxides mediated by CYP2C37, CYP2C50, and CYP2C54 (Fig 4). However, we believe that more research is needed to understand the link between them.

We also showed the down-regulation of *Magl* and *Fabp4* expression levels in the liver of SNL-fed *db*/*db* mice. The effect of dietary EPA/DHA on *Magl* expression has been described with high fat diet (HFD)-fed mice; supplementation with EPA/DHA to HFD resulted in trend toward reduced *Magl* expression levels in adipose tissue [30]. These previous findings suggest that EPA elevated in liver of SNL-fed *db*/*db* mice may contribute to *Magl* down-regulation. MAGL supply the AA as precursor of eicosanoids in liver and lung, in contrast to cPLA_2_ which regulate these lipids in gut and spleen (Fig 4) [31]. In fact, we detected *Magl* expression, but not *cPla*_*2*_, in the liver of all tested mice, implicating the importance of MAGL in liver of *db*/*db* mice. Furthermore, EPA or DHA treatment also decrease the serum FABP4 concentration in patients with dyslipidemia [32]. In addition, 3T3-L1 adipocytes treated with EPA or DHA displayed a significant decrease of *Fabp4* expression and protein secretion [32]. These findings show that *Fabp4* down-regulation found in SNL-fed *db*/*db* mice also might be caused by EPA released from SNL.

The accumulation of hepatic LA, GLA, DGLA, and AA detected in *db*/*db* mice was attenuated in SNL-fed *db*/*db* mice. Our findings indicate that the alterations in the expression levels of AA and LA metabolism-related genes induced by the SNL diet may contribute to the reduction in these 4 fatty acids (Fig 4). Nevertheless, the concentration of AA appeared to differ from the other 3 fatty acids, as we observed an unexpectedly low level of AA in the liver of *db*/*db* mice. Previous studies have established that the desaturation process of LA is impaired in insulin-dependent diabetic rats, resulting in decreased AA levels in various tissues [33, 34]. Furthermore, *db*/*db* mice treated with Ex-4, a glucagon-like peptide-1 receptor (GLP-1R) agonist implicated in the development of NASH, exhibited a decrease in D5D activity and elevated levels of LA, GAL, and DGLA, but not AA [35]. In fact, we detected significant down-regulation of *Fads1* and *Fads2* in our *db*/*db* mice comparing to the normal mice, suggesting a decrease in D5D and D6D activities. Thus, the impairment in the hepatic desaturation steps of LA in our *db*/*db* mice may contribute to the reduction in hepatic AA concentration. Furthermore, we observed that SNL-fed *db*/*db* mice demonstrated even lower AA levels than those found in *db*/*db* mice, but exhibited an excessive accumulation of EPA. Interestingly, the dietary supplementation of EPA/DHA resulted in a significant replacement of AA by EPA/DHA in the liver [24]. These findings imply that the replacement of AA by EPA released from SNL may also contribute to the reduction of AA.

Other than AA and LA metabolism-related genes, 15 SNL-dependent up-regulated genes included *Gpld1* and *Scarb1*, which contributed to a significant enrichment of the term “Positive regulation of triglyceride biosynthetic process” in our BP GO analysis. GPLD1, also known as glycosylphosphatidylinositol (GPI)‐specific phospholipase D (GPI‐PLD), is one of high-density lipoprotein (HDL)-associated protein [36, 37]. The serum concentration of GPLD1 is directly proportional to total triglyceride, cholesterol, and insulin, implying a pivotal role for GPLD1 in triglyceride metabolism, insulin resistance, and the development of diabetes [38]. Interestingly, an increase in GPLD1 and a decrease in plasma triglycerides have been observed in normolipidemic adults supplemented with EPA- or DHA-rich fish oil [39]. Furthermore, overexpression of *Gpld1* has been shown to affect the expression levels of some genes related to fatty acid and lipid metabolism, including *Scarb1* [40]. *Scarb1* encodes HDL receptor known as scavenger receptor class B type I (SR-BI), which mediates cholesterol flux from HDL into the liver [41]. As hepatic SR-BI expression is regulated by leptin, the down-regulation of SR-BI detected in leptin-deficient mice is thought to be an important explanatory mechanism for elevated HDL cholesterol levels [42]. SNL diet have been reported to decrease the levels of hepatic triglyceride and serum cholesterol in *db*/*db* mice [7]. We propose that the up-regulation of *Gpld1* and *Scarb1* might contribute to these SNL-induced phenotypic alterations (Fig 4).

Additionally, we anticipated that *Hal* and *Amdhd1*, which contributed to a significant enrichment of histidine-related pathway and GO terms in our RNA-Seq analysis. HAL and AMDHD1 are involved in the degradation pathway of histidine, a dietary essential amino acid for cellular growth and proliferation. Our results suggest that the histidine degradation pathway may be suppressed in the liver of *db*/*db* mice, but is reactivated by SNL diet. Histidine is known as a precursor for the synthesis of histamine. Interestingly, histamine has been shown to acts as an anorexigenic agent by mediating the effects of leptin [43, 44]. Recent work has demonstrated that disrupting histamine/leptin signaling can prevent cholangiocyte damage and hepatic fibrosis in HFD-fed mice [45]. Based on these findings from previous studies and our results, it is possible that changes in histidine levels caused by the SNL diet could influence histamine/leptin signaling and lead to the improvement of hepatic steatosis.

## Conclusion

To provide a comprehensive understanding of the SNL diet, we have reported here the first analysis of the genome-wide impact on the livers of *db*/*db* mice. The transcriptome analyses conducted in this study identify the 15 SNL-dependent up-regulated genes, and subsequent experiments have demonstrated their significant association to AA and LA metabolisms. Our results, as well as previous research, suggest that changes in gene expression are affected by the EPA, which is primarily released from SNL. The alterations in the expression of these 15 genes may contribute to the beneficial effects of SNL on hepatic steatosis, either directly or indirectly.

Our findings enhance the efficacy of SNL as a dietary supplement to ameliorate hepatic steatosis. The 15 genes identified in this study could potentially serve not only as targets for SNL but also as the novel candidate molecules for NAFLD therapy. In Japan, Susabinori of poor food quality can sometimes be discarded. Turning Susabinori into a cost-effective source of SNL, which is rich in EPA, without discarding it, is a challenge for the future.

## Supporting information

S1 Fig Comparison of RNA-Seq results with those of qRT-PCR. The Log_2_FC values of randomly selected 17 genes (gray bar), including SNL-dependent 9 up- or 8 down-regulated genes, were compared to the results obtained from qRT-PCR (white bar). The Log_2_FC values were obtained by DEGs calculation between control and SNL groups. The values of qRT-PCR were calculated from the results of three independent experiments, and presented Means ± standard errors.
(TIF)

S1 Table Primer sets used for quantitative RT-PCR (qRT-PCR). (DOCX)

S2 Table EPA contents in organs and serum. (DOCX)

## References

[1] Smith BW, Adams LA. Non-alcoholic fatty liver disease. Crit Rev Clin Lab Sci. 2011;48(3):97–113. doi: 10.3109/10408363.2011.596521 21875310

[2] Pouwels S, Sakran N, Graham Y, Leal A, Pintar T, Yang W, et al. Non-alcoholic fatty liver disease (NAFLD): a review of pathophysiology, clinical management and effects of weight loss. BMC Endocr Disord. 2022;22: 1–9. 35287643 10.1186/s12902-022-00980-1 PMC8919523

[3] Youssef W, McCullough AJ. Diabetes mellitus, obesity, and hepatic steatosis. Semin Gastrointest Dis. 2002;13: 17–30. 11944630

[4] Powell EE, Wong VWS, Rinella M. Non-alcoholic fatty liver disease. Lancet Lond Engl. 2021;397(10290): 2212–24. 10.1016/S0140-6736(20)32511-3 33894145

[5] Yang LE, Deng YY, Xu GP, Russell S, Lu QQ, Brodie J. Redefining Pyropia (Bangiales, Rhodophyta): Four New Genera, Resurrection of *Porphyrella* and Description of *Calidia pseudolobata* sp. nov. From China. J Phycol. 2020;56(4): 862–79. 32196675 10.1111/jpy.12992

[6] Zuccarello GC, Wen X, Kim GH. Splitting blades: why genera need to be more carefully defined; the case for *Pyropia* (Bangiales, Rhodophyta). Algae. 2022;37(3): 205–11.

[7] Yanagita T, Tsuge K, Koga M, Inoue N, Nagao K. Eicosapentaenoic acid-containing polar lipids from seaweed Susabinori (*Pyropia yezoensis*) alleviate hepatic steatosis in obese *db*/*db* mice. Arch Biochem Biophys. 2020;691: 108486. 32710880 10.1016/j.abb.2020.108486

[8] Folch J, Lees M, Slonane Stanley GH. A simple method for the isolation and purification of total lipides from animal tissues. J Biol Chem. 1957;226: 497–509. 13428781

[9] Bligh EG, Dyer WJ. A rapid method of total lipid extraction and purification. Can J Biochem Physiol. 1959;37(8): 911–7. doi: 10.1139/o59-099 13671378

[10] Sun J, Nishiyama T, Shimizu K, Kadota K. TCC: an R package for comparing tag count data with robust normalization strategies. BMC Bioinformatics. 2013;14: 219. doi: 10.1186/1471-2105-14-219 23837715 PMC3716788

[11] Robinson MD, McCarthy DJ, Smyth GK. edgeR: a Bioconductor package for differential expression analysis of digital gene expression data. Bioinformatics. 2010;26:139–40. doi: 10.1093/bioinformatics/btp616 19910308 PMC2796818

[12] Huang DW, Sherman BT, Lempicki RA. Systematic and integrative analysis of large gene lists using DAVID bioinformatics resources. Nat Protoc. 2009;4: 44–57. doi: 10.1038/nprot.2008.211 19131956

[13] Kanehisa M, Goto S, Kawashima S, Nakaya A. The KEGG databases at GenomeNet. Nucleic Acids Res. 2002;30: 42–6. doi: 10.1093/nar/30.1.42 11752249 PMC99091

[14] Heller A, Koch T, Schmeck J, van Ackern K. Lipid Mediators in Inflammatory Disorders. Drugs. 1998;55(4): 487–96. doi: 10.2165/00003495-199855040-00001 9561339 PMC7102224

[15] Tapiero H, Nguyen Ba G, Couvreur P, Tew KD. Polyunsaturated fatty acids (PUFA) and eicosanoids in human health and pathologies. Biomed Pharmacother. 2002;56(5): 215–22. doi: 10.1016/s0753-3322(02)00193-2 12199620

[16] Arnold C, Konkel A, Fischer R, Schunck WH. Cytochrome P450-dependent metabolism of ω-6 and ω-3 long-chain polyunsaturated fatty acids. Pharmacol Rep. 2010;62(3): 536–47. 20631419 10.1016/s1734-1140(10)70311-x

[17] Luo G, Zeldin DC, Blaisdell JA, Hodgson E, Goldstein JA. Cloning and Expression of Murine CYP2Cs and Their Ability to Metabolize Arachidonic Acid. Arch Biochem Biophys. 1998;357: 45–57. doi: 10.1006/abbi.1998.0806 9721182

[18] Wang H, Zhao Y, Bradbury JA, Graves JP, Foley J, Blaisdell JA, et al. Cloning, Expression, and Characterization of Three New Mouse Cytochrome P450 Enzymes and Partial Characterization of Their Fatty Acid Oxidation Activities. Mol Pharmacol. 2004;65(5): 1148–58. doi: 10.1124/mol.65.5.1148 15102943

[19] Isobe Y, Itagaki M, Ito Y, Naoe S, Kojima K, Ikeguchi M, et al. Comprehensive analysis of the mouse cytochrome P450 family responsible for omega-3 epoxidation of eicosapentaenoic acid. Sci Rep. 2018;8: 7954. doi: 10.1038/s41598-018-26325-4 29784972 PMC5962638

[20] Wang W, Yang J, Yang H, Sanidad KZ, Hammock BD, Kim D, et al. Effects of high-fat diet on plasma profiles of eicosanoid metabolites in mice. Prostaglandins Other Lipid Mediat. 2016;127: 9–13. doi: 10.1016/j.prostaglandins.2016.11.003 27913146 PMC5628263

[21] Luo P, Wang MH. Eicosanoids, β-cell function, and diabetes. Prostaglandins Other Lipid Mediat. 2011;95: 1–10. 21757024 10.1016/j.prostaglandins.2011.06.001 PMC3144311

[22] Maciejewska D, Drozd A, Skonieczna-Żydecka K, Skórka-Majewicz M, Dec K, Jakubczyk K, et al. Eicosanoids in Nonalcoholic Fatty Liver Disease (NAFLD) Progression. Do Serum Eicosanoids Profile Correspond with Liver Eicosanoids Content during NAFLD Development and Progression? Molecules. 2020;25(9): 2026. doi: 10.3390/molecules25092026 32349225 PMC7248881

[23] Xu X, Zhao CX, Wang L, Tu L, Fang X, Zheng C, et al. Increased *CYP2J3* Expression Reduces Insulin Resistance in Fructose-Treated Rats and *db*/*db* Mice. Diabetes. 2010;59(4): 997–1005. 20068141 10.2337/db09-1241 PMC2844847

[24] Arnold C, Markovic M, Blossey K, Wallukat G, Fischer R, Dechend R, et al. Arachidonic Acid-metabolizing Cytochrome P450 Enzymes Are Targets of ω-3 Fatty Acids. J Biol Chem. 2010;285(43): 32720–33. 20732876 10.1074/jbc.M110.118406 PMC2963419

[25] Node K, Huo Y, Ruan X, Yang B, Spiecker M, Ley K, et al. Anti-inflammatory Properties of Cytochrome P450 Epoxygenase-Derived Eicosanoids. Science. 1999;285(5431): 1276–9. doi: 10.1126/science.285.5431.1276 10455056 PMC2720027

[26] Yang S, Lin L, Chen JX, Lee CR, Seubert JM, Wang Y, et al. Cytochrome *P-450* epoxygenases protect endothelial cells from apoptosis induced by tumor necrosis factor-α via MAPK and PI3K/Akt signaling pathways. Am. J. Physiol. Heart Circ. Physiol. 2007;293: H142–51. 17322420 10.1152/ajpheart.00783.2006 PMC2100428

[27] Chen G, Xu R, Zhang S, Wang Y, Wang P, Edin ML, et al. CYP2J2 overexpression attenuates nonalcoholic fatty liver disease induced by high-fat diet in mice. Am. J. Physiol. Endocrinol. Metab. 2015;308: E97–110. doi: 10.1152/ajpendo.00366.2014 25389366 PMC4297779

[28] López-Vicario C, Alcaraz-Quiles J, García-Alonso V, Rius B, Hwang SH, Titos E, et al. Inhibition of soluble epoxide hydrolase modulates inflammation and autophagy in obese adipose tissue and liver: Role for omega-3 epoxides. Proc Natl Acad Sci. 2015;112: 536–41. doi: 10.1073/pnas.1422590112 25550510 PMC4299190

[29] Warner D, Vatsalya V, Zirnheld KH, Warner JB, Hardesty JE, Umhau JC, et al. Linoleic Acid‐Derived Oxylipins Differentiate Early Stage Alcoholic Hepatitis From Mild Alcohol‐Associated Liver Injury. Hepatol Commun. 2021;5: 947–60. doi: 10.1002/hep4.1686 34141982 PMC8183177

[30] Rossmeisl M, Pavlisova J, Janovska P, Kuda O, Bardova K, Hansikova J, et al. Differential modulation of white adipose tissue endocannabinoid levels by n-3 fatty acids in obese mice and type 2 diabetic patients. Biochim Biophys Acta (BBA)- Mol Cell Biol Lipids. 2018;1863: 712–25. doi: 10.1016/j.bbalip.2018.03.011 29626526

[31] Nomura DK, Morrison BE, Blankman JL, Long JZ, Kinsey SG, Marcondes MCG, et al. Endocannabinoid Hydrolysis Generates Brain Prostaglandins That Promote Neuroinflammation. Science. 2011;334(6057): 809–13. doi: 10.1126/science.1209200 22021672 PMC3249428

[32] Furuhashi M, Hiramitsu S, Mita T, Omori A, Fuseya T, Ishimura S, et al. Reduction of circulating FABP4 level by treatment with omega-3 fatty acid ethyl esters. Lipids in Health and Disease. 2016;15(5). doi: 10.1186/s12944-016-0177-8 26754658 PMC4710044

[33] Mercuri O, Peluffo RO, Brenner RR. Depression of microsomal desaturation of linoleictoy-linolenicacid in the alloxan-diabetic rat. Biochim Biophys Acta (BBA)- Lipids Lipid Metab. 1966;116: 409–11. 5956925

[34] Cunnane SC, Manku MS, Horrobin DF. Abnormal essential fatty acid composition of tissue lipids in genetically diabetic mice is partially corrected by dietary linoleic and γ-linolenic acids. Br J Nutr. 1985;53: 449–58. 2998444 10.1079/bjn19850054

[35] Kawaguchi T, Itou M, Taniguchi E, Sata M. Exendin‑4, a glucagon‑like peptide‑1 receptor agonist, modulates hepatic fatty acid composition and Δ‑5‑desaturase index in a murine model of non‑alcoholic steatohepatitis. Int J Mol Med. 2014;34: 782–7 24993337 10.3892/ijmm.2014.1826

[36] Hoener MC, Brodbeck U. Phosphatidylinositol-glycan-specific phospholipase D is an amphiphilic glycoprotein that in serum is associated with high-density lipoproteins. Eur J Biochem. 1992;206: 747–57. doi: 10.1111/j.1432-1033.1992.tb16981.x 1606959

[37] Deeg MA, Bierman EL, Cheung MC. GPI-specific phospholipase D associates with an apoA-I- and apoA-IV-containing complex. J Lipid Res. 2001;42: 442–51. 11254757

[38] Kurtz TA, Fineberg NS, Considine RV, Deeg MA. Insulin resistance is associated with increased serum levels of glycosylphosphatidylinositol-specific phospholipase D. Metabolism. 2004;53: 138–9. doi: 10.1016/j.metabol.2003.09.004 14767861

[39] Yang ZH, Amar M, Sampson M, Courville AB, Sorokin AV, Gordon SM, et al. Comparison of Omega-3 Eicosapentaenoic Acid Versus Docosahexaenoic Acid-Rich Fish Oil Supplementation on Plasma Lipids and Lipoproteins in Normolipidemic Adults. Nutrients. 2020;12: 749. doi: 10.3390/nu12030749 32178279 PMC7146314

[40] Chalasani N, Vuppalanchi R, Raikwar NS, Deeg MA. Glycosylphosphatidylinositol-Specific Phospholipase D in Nonalcoholic Fatty Liver Disease: A Preliminary Study. J Clin Endocrinol Metab. 2006;91(6): 2279–85. doi: 10.1210/jc.2006-0075 16595594

[41] Acton S, Rigotti A, Landschulz KT, Xu S, Hobbs HH, Krieger M. Identification of Scavenger Receptor SR-BI as a High Density Lipoprotein Receptor. Science. 1996;271(5248): 518–20. doi: 10.1126/science.271.5248.518 8560269

[42] Lundåsen T, Liao W, Angelin B, Rudling M. Leptin Induces the Hepatic High Density Lipoprotein Receptor Scavenger Receptor B Type I (SR-BI) but Not Cholesterol 7α-Hydroxylase (Cyp7a1) in Leptin-deficient (ob/ob) Mice. J Biol Chem. 2003;278(44): 43224–8. 12917427 10.1074/jbc.M302645200

[43] Itateyama E, Chiba S, Sakata T, Yoshimatsu H. Hypothalamic Neuronal Histamine in Genetically Obese Animals: Its Implication of Leptin Action in the Brain. Exp Biol Med. 2003;228(10): 1132–7. doi: 10.1177/153537020322801006 14610251

[44] Yoshimatsu H. Hypothalamic neuronal histamine regulates body weight through the modulation of diurnal feeding rhythm. Nutrition. 2008;24(9): 827–31. doi: 10.1016/j.nut.2008.06.014 18725079

[45] Kennedy L, Hargrove L, Demieville J, Bailey JM, Dar W, Polireddy K, et al. Knockout of l-Histidine Decarboxylase Prevents Cholangiocyte Damage and Hepatic Fibrosis in Mice Subjected to High-Fat Diet Feeding via Disrupted Histamine/Leptin Signaling. Am J Pathol. 2018;188(3): 600–15. doi: 10.1016/j.ajpath.2017.11.016 29248461 PMC5840487

