# Supplementary figures and images for: Identification of genes regulated by lipids from seaweed Susabinori (Pyropia yezoensis) involved in the improvement of hepatic steatosis: Insights from RNA-Seq analysis in obese db/db mice
Source: PLoS One. 2023 Dec 12;18(12):e0295591. doi: 10.1371/journal.pone.0295591 (PMC10715663; doi:10.1371/journal.pone.0295591)

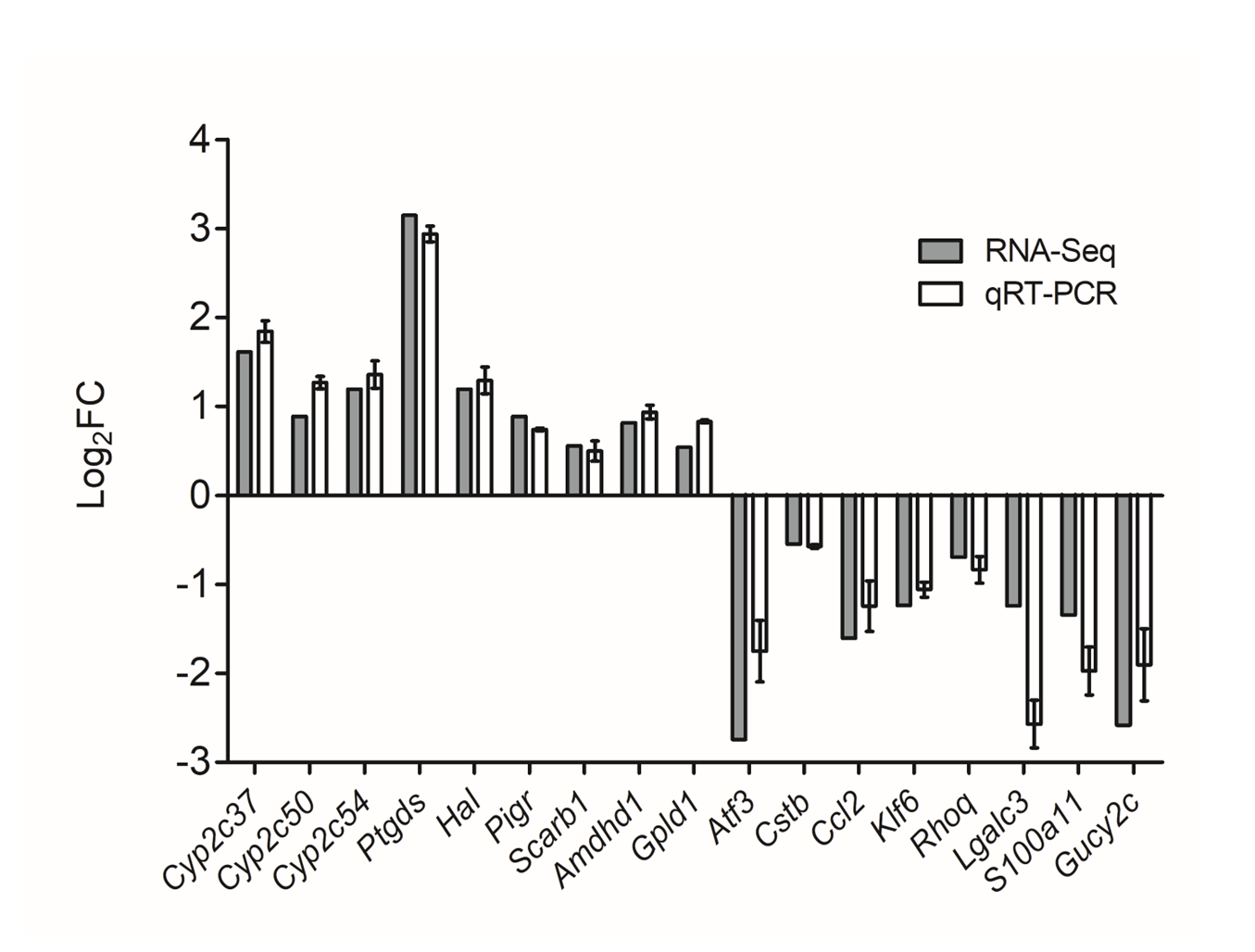

Supplement: S1 Fig — The Log2FC values of randomly selected 17 genes (gray bar), including SNL-dependent 9 up- or 8 down-regulated genes, were compared to the results obtained from qRT-PCR (white bar). The Log2FC values were obtained by DEGs calculation between control and SNL groups. The values of qRT-PCR were calculated from the results of three independent experiments, and presented Means ± standard errors. (TIF) [file pone.0295591.s001.tif]
