# Supplementary material for: Identification of genes regulated by lipids from seaweed Susabinori (Pyropia yezoensis) involved in the improvement of hepatic steatosis: Insights from RNA-Seq analysis in obese db/db mice
Source: PLoS One. 2023 Dec 12;18(12):e0295591. doi: 10.1371/journal.pone.0295591 (PMC10715663; doi:10.1371/journal.pone.0295591)
Supplement: S1 Table — (DOCX) [file pone.0295591.s002.docx]

**S1 Table.** **Primer sets used for quantitative RT-PCR (qRT-PCR).**

| Gene | F/R | Sequence |
| --- | --- | --- |
| *Ptgds* | F | 5’- CACAGAGGAGGACATTGTTTTCC -3’ |
|  | R | 5’- ACTGACTTCTCTCACCTGCGTTT -3’ |
| *Cyp2c37* | F | 5’- AGATGGCAATCAACCATTGCAAAA -3’ |
|  | R | 5’- TGGACTTTAGCTGTGACATGTGG -3’ |
| *Cyp2c50* | F | 5’- GCTGTGCTCCCTGCAATGTC -3’ |
|  | R | 5’- TGCAAACCTGCAACCAAGGG -3’ |
| *Cyp2c54* | F | 5’- AAGGAGCCCAGGAAGATGACAATCA -3’ |
|  | R | 5’- GCTCATTGACTCTGTCCCACCAATA -3’ |
| *Hal* | F | 5’- CCTGGTGCGCTCCGTAGTAA -3’ |
|  | R | 5’- CGGCAACCTCCCAAACCTTTT -3’ |
| *Pigr* | F | 5’- CAGGTTGCCGAAGCTACAAGG -3’ |
|  | R | 5’- TTGCACGGATAGTGGCAGGAA -3’ |
| *Scarb1* | F | 5’- GCAAGAAGCCAAGCTATAGGGTC -3’ |
|  | R | 5’- AGTCCTCAAGAAGCGGGGTG -3’ |
| *Amdhd1* | F | 5’- CGTACTGCTTTTCCATGCCGA -3’ |
|  | R | 5’- ATAAGCCGCGTTGATGGTGG -3’ |
| *Gpld1* | F | 5’- TGAGGTGAGGATATTGGAGATGC -3’ |
|  | R | 5’- ACTGTCCACCTATGCCAGTTGA -3’ |
| *Atf3* | F | 5’- ACAGAGTGCCTGCAGAAAGAGT -3’ |
|  | R | 5’- CCATTCTGAGCCCGGACGAT -3’ |
| *Cstb* | F | 5’- CTTCTCCGTGCTACCCCGAC -3’ |
|  | R | 5’- TTCAAGCTGGGACTTCACCTGG -3’ |
| *Ccl2* | F | 5’- AAGCTGTAGTTTTTGTCACCAAGC -3’ |
|  | R | 5’- GACCTTAGGGCAGATGCAGT -3’ |
| *Klf6* | F | 5’- CAGCGCACTCACACAGGAGAA -3’ |
|  | R | 5’- TGGCACCGGTATGCTTTCGG -3’ |
| *Rhoq* | F | 5’- TCTTCGACCACTACGCAGTCAG -3’ |
|  | R | 5’- GGAAGACGTCAGTCATGGGGT -3’ |
| *Lgals3* | F | 5’- CACAGAGAGCACTACCCAGGAAA -3’ |
|  | R | 5’- TTCCCCCATGCACCCGGATA -3’ |
| *S100a11* | F | 5’- GGCTGCCTTCACAAAGAACCAG -3’ |
|  | R | 5’- CTGCCCGTCACAGTTGAGGT -3’ |
| *Gucy2c* | F | 5’- GCCTGAAGATTGACGATGACAGG -3’ |
|  | R | 5’- ACTGAAGTTCCCGTCGCTGT -3’ |
| *Magl* | F | 5’- TCGGAACAAGTCGGAGGT -3’ |
|  | R | 5’- TCAGCAGCTGTATGCCAAAG -3’ |
| *Fads1* | F | 5’- TGTGTGGGTGACACAGATGA -3’ |
|  | R | 5’- GTTGAAGGCTGATTGGTGAA -3’ |
| *Fads2* | F | 5’- CCACCGACATTTCCAACAC -3’ |
|  | R | 5’- GGGCAGGTATTTCAGCTTCTT -3’ |
| *Fabp4* | F | 5’- CTGGCCAACATCAACTTCAG -3’ |
|  | R | 5’- GCCAGTCTCTTCTGCATAGT -3’ |
| *Pparγ* | F | 5’- CAAGAATACCAAAGTGCGATCAA -3’ |
|  | R | 5’- GAGCTGGGTCTTTTCAGAATAATAAG -3’ |
| *β-actin* | F | 5’- GGTGGGAATGGGTCAGAAGG -3’ |
|  | R | 5’- GTACATGGCTGGGGTGTTG -3’ |
