# Supplementary material for: Identification of genes regulated by lipids from seaweed Susabinori (Pyropia yezoensis) involved in the improvement of hepatic steatosis: Insights from RNA-Seq analysis in obese db/db mice
Source: PLoS One. 2023 Dec 12;18(12):e0295591. doi: 10.1371/journal.pone.0295591 (PMC10715663; doi:10.1371/journal.pone.0295591)
Supplement: S2 Table — (DOCX) [file pone.0295591.s003.docx]

**S2 Table. EPA contents in organs and serum.**

|  | (mg/mg tissue) | | | | | | | | | (mg/ml) |
| --- | --- | --- | --- | --- | --- | --- | --- | --- | --- | --- |
|  | **Liver** | **Brain** | **Heart** | **Spleen** | **Kidney** | **AdrenalGrand** | **Testis** | **Muscle** | **WAT** | **Serum** |
| **Normal** | 0.010±0.000 | 0.010±0.000 | 0.003±0.003 | ND | 0.018±0.004 | 0.297±0.059 | 0.005±0.002 | 0.000 | ND | ND |
| **Control** | 0.035±0.005 | 0.000 | 0.033±0.002 | 0.002±0.002 | 0.092±0.007 | 0.233±0.032 | 0.002±0.002 | 0.000 | ND | 0.020±0.003 |
| **SNL** | 1.908±0.146 | 0.117±0.008 | 0.105±0.022 | 0.847±0.017 | 2.690±0.065 | 3.443±0.457 | 0.098±0.003 | 0.300±0.045 | 4.295±1.326 | 0.570±0.033 |

The contents of EPA in liver, brain, heart, spleen, kidney, adrenal grand, testis, muscle, white adipose tissue from perirenal (WAT), and serum were measured (n = 6). Means ± standard errors are presented. “ND” indicate no detectable organs or serum.
